# Optimizing Sensory Experience and Aroma Profile Through Novel Blending: A Dual GC‐MS/GC‐IMS Approach to Characterize *Nitraria tangutorum* Bobrov.–Blended Wine

**DOI:** 10.1155/ijfo/3378651

**Published:** 2026-04-29

**Authors:** Hao Chen, Ying Li, Ming Chi, Yuting Wang, Numan Khan, Ni Yang, Xuefei Wang, Yanzhen Zhang, Fei Wang, Yuhua Bao, Yulin Zhang, Zhumei Xi

**Affiliations:** ^1^ College of Enology, Northwest A&F University, Yangling, Xianyang, Shaanxi, 712100, China, nwsuaf.edu.cn; ^2^ Guizhou Xijiu Co., Ltd, Zunyi, Guizhou, 564622, China; ^3^ Qinghai Light Industry Institute Co., Ltd, Xining, Qinghai, 810008, China

**Keywords:** aroma, GC-IMS, GC-MS, *Nitraria tangutorum* Bobrov

## Abstract

*Nitraria tangutorum* Bobrov. (*N. tangutorum*) is a drought‐tolerant and salt‐loving plant with high nutrient value, native to northwest China. Its distribution in the Qaidam Basin covers nearly 1000 square kilometers, yielding 0.5–10 × 10^5^ tons of industrial juice by‐products annually. However, the bitter and fishy taste of the plant limits its industrial application. Blending is an effective technique to enhance the sensory qualities of wine. In this study, eight blended wines were prepared by mixing *N. tangutorum*–fermented wine (NT) with either Meili rosé wine (ML) or a combination of highland barley wine (QK) and “Shine Muscat” grape juice (SM) in different proportions. The results showed that both blending methods significantly reduced titratable acidity and volatile acidity of NT, while increasing its flavanol and tannin contents as well as brightness. Sensory evaluation revealed that the blended samples NT‐QK‐SM‐2 (30%:35%:35%) and NT‐ML‐2 (15%:85%) received higher scores in appearance, aroma, taste, and overall quality. Analyses by gas chromatography‐ion mobility spectrometry (GC‐IMS) and GC‐mass spectrometry (GC‐MS) indicated that NT‐QK‐SM‐2 contained higher levels of ethyl lactate, 2‐methyl‐1‐propanol, 1‐butanol, and other compounds, contributing to its unique aromatic profiles. In contrast, NT‐ML‐2 showed elevated concentrations of acetaldehyde, isobutyl acetate, and other compounds, imparting a more pronounced floral and fruity flavor. Partial least squares discriminant analysis (PLS‐DA) identified ten key biomarkers for distinguishing the blended wines: ethyl caproate, ethyl butyrate, ethyl isobutyrate, octanal, ethyl succinate, β‐ionone, 2‐methyl‐4‐ethylphenol, (E)‐3‐hexen‐1‐ol, 1‐hexanol, and 1‐propanol. These findings demonstrate that blending can significantly improve the aroma and sensory quality of *N. tangutorum* wine, offering a viable approach to transform this nutrient‐rich but otherwise single‐flavored resource into a product with market potential.

## 1. Introduction

*Nitraria tangutorum*​ Bobrov. is a perennial deciduous shrub of the genus *Nitraria* within the family Nitrariaceae [1]. It is widely distributed across the desert and semidesert saline–alkaline sandy lands of the Qaidam Basin on the Qinghai–Tibetan Plateau [2]. This species exhibits high resilience to environmental stresses, particularly drought, high salinity, and alkaline conditions [3]. Although small, its berries are rich in bioactive compounds such as flavonoids, anthocyanins, amino acids, polysaccharides, vitamins, organic acids, minerals, and trace elements [2]. In the Qaidam Basin alone, *N. tangutorum* covers an area of nearly 1000 km^2^, yielding an estimated 0.5–1.0 × 10^5^ tons of industrial juice by‐products annually from its berries [3, 4]. The juice is characterized by a high sugar content and low acidity, making it suitable for producing a variety of foods and beverages, including jams, biscuits, jelly, soft candies, and fermented drinks. The development of fermented beverages from *N. tangutorum* not only diversifies consumer choices but also extends the industry chain and enhances economic value. However, fermented beverages derived from *N. tangutorum* often suffer from sensory limitations such as a bitter, fishy taste, and insufficient aroma [5]. Therefore, exploring new processing techniques to improve the aromatic and sensory profile of *N. tangutorum*–fermented beverage remains necessary.

Blending is a well‐established winemaking technique that combines different wines to achieve improved flavor profiles, balance, and complexity. The resulting blended wines exhibit desirable sensory properties and appearance, meeting the global demand for diversified wine products [6, 7]. For instance, Xu et al. [8] reported that blending pear and Merlot grape alcoholic beverages increased terpene and ethyl ester contents by 16.5% and 11.2%, respectively, leading to significantly enhanced floral and fruity aromas and higher sensory scores. Similarly, blending apple or Merlot wines with pear beverages have been shown to elevate the concentrations of ethyl esters, higher alcohols, terpenes, and C13‐norisoprenoids compared to pure pear beverages [9, 10]. In contrast, research on the fermentation of prickly fruit such as *N. tangutorum* remains limited. Previous studies have focused primarily on optimizing fermentation parameters, such as yeast inoculation and enzymatic hydrolysis time, yet these adjustments have not fully resolved issues related to bitter taste, fishy odor, and poor clarification [11, 12]. Blending offers a promising approach to mitigating such challenges by modifying chemical composition, adjusting color, and enhancing the complexity of the wine body and aroma [13]. Moreover, in contexts where grape crops face challenges such as inconsistent coloration or overproduction [14], blending with other fruit wines like those from *N. tangutorum* could provide a viable utilization pathway. Therefore, applying blending techniques to *N. tangutorum* wine may not only ameliorate its bitterness and fishy taste but also optimize its color and overall sensory profile. This strategy could enhance the utilization efficiency of prickly fruit resources while concurrently offering a potential solution to grape oversupply.

In recent years, gas chromatography‐ion mobility spectrometry (GC‐IMS) has gained widespread use in the detection of volatile compounds in foods [15]. This technology effectively couples the high separation capability of GC with the rapid response and high sensitivity of IMS, enabling the detection of a broad range of compounds across diverse chemical groups [16]. Zhu et al. [17] were among the first to employ GC‐IMS for wine aroma analysis, identifying volatiles such as methyl acetate and ethyl formate that had rarely been reported in Sauvignon Blanc wines. Liu et al. [18] established a flavor fingerprint for cherry wine using GC‐IMS combined with principal component analysis (PCA), providing a theoretical foundation for evaluating and improving flavor quality during cherry wine fermentation. Similarly, Tang and Peng [19] applied GC‐MS and GC‐IMS to elucidate flavor distinctions between black rice wine and glutinous rice wine. Despite the demonstrated utility of GC‐IMS in food analysis, however, research targeting the volatile composition of *N. tangutorum* wines remains limited, with few studies systematically characterizing their aroma profiles using this technique.

*N. tangutorum* thrives in saline–alkali desert environments, leading its berries to absorb inorganic salt ions (e.g., Na^+^ and K^+^) from the soil, which can impart a noticeable salty taste to the resulting fruit wine. During fermentation, pronounced bitterness and fishy odors often develop, negatively affecting the product’s flavor and mouthfeel. To overcome these drawbacks and improve consumer acceptance, wine blending has been adopted to modify key attributes such as color, flavor, alcohol content, body, and aromatic profile, thereby enhancing overall quality. Against the backdrop of growing global demand for health‐oriented beverages with distinctive flavors and nutritional benefits, developing blended *N. tangutorum* wine represents a promising opportunity to align with market trends and support the local agricultural economy. In this study, the influence of different blending methods on the volatile compounds in *N. tangutorum* wine was investigated using GC‐MS. Additionally, GC‐IMS was employed to obtain volatile compound fingerprints for each blending formulation. The physicochemical properties of the wines prepared with different blending ratios were also systematically evaluated. The results provide a theoretical foundation for improving the flavor and quality of *N. tangutorum* wine and open new avenues for innovative product development in the beverage industry.

## 2. Materials and Methods

### 2.1. Fruit and Wine Materials

Berries of *N. tangutorum* were collected from Delingha, Qinghai, China (37°13′N, 97°14′E, altitude 2980 m) and transported on ice. The collection site features large diurnal temperature variations, an arid climate, long sunshine duration, strong solar radiation, and frequent sandstorms. The mean annual temperature ranges from 7.8°C to 8.1°C, with annual precipitation totaling 162–172 mm concentrated mainly between June and August. Ripe grapes of “Meili” (*Vitis vinifera* L.) and “Shine Muscat” (*V. labruscana*) were harvested from a vineyard in Yangling, Shaanxi, China (108°72′E, 34°36′N). The region experiences a continental monsoon climate with distinct seasons, moderate diurnal temperature variation, and semihumid conditions, along with long sunshine hours and considerable solar radiation. The average annual temperature is about 12.9°C, and annual precipitation totals 635–663 mm. Highland barley wine was supplied by Tianyoude Qingke Wine Co., Ltd., Haidong City, Qinghai Province, China. The soluble solids and titratable acids of the three raw materials prior to fermentation are shown in Table S1.

### 2.2. Wine Making and Blending

*N. tangutorum* and Meili berries were separately destemmed and crushed. Winemaking was carried out following a laboratory‐scale microvinification procedure adapted from Gao et al. [20]. Immediately after crushing, sulfur dioxide was added at 60 mg/L, followed by pectinase (BXL, Gestown, China) at 30 mg/L. The must was then inoculated with 200 mg/L *Saccharomyces cerevisiae* (Erbslöh, Germany) to initiate alcoholic fermentation. The density (measured by hydrometer) and temperature of the fermenting must were monitored three times per day until sugar depletion. Fermentation was stopped by adding 60 mg/L SO_2_, after which the skins were pressed off. The wines underwent cold stabilization at 4 ± 1°C for three months, were filtered through a 0.22‐μm membrane, bottled, and stored in a cellar at 20 ± 3°C. The entire vinification process was performed in duplicate for each wine sample.

*N. tangutorum*–fermented wine (NT) was blended with Meili rosé wine (ML) in volumetric ratios (NT:ML): 10:90, 15:85, 20:80, and 25:75. Separately, NT was blended with highland barley wine (QK) and Shine Muscat juice (SM) at the volumetric ratios (NT:QK:SM) of 30:30:40, 30:35:35, 50:30:20, and 50:25:25 (Table 1). After blending, the free sulfur dioxide level in each wine was adjusted to 30 mg/L. All wines were then immediately bottled, sealed with cork, and stored at 10°C until further analysis.

**TABLE 1:** The complex wine ratio of *N. tangutorum*–blended fruit wine.

Type	Number	Proportion
Meili rosé blended with *N. tangutorum* wine	CK	*N. tangutorum* wine = 100%
	ML	Meili rosé wine = 100%
	QK‐SM	Barley wine: “Shine Muscat” grape juice = 50%:50%
	NT‐ML‐1	*N. tangutorum* wine: Meili rosé wine = 10%:90%
	NT‐ML‐2	*N. tangutorum* wine: Meili rosé wine = 15%:85%
	NT‐ML‐3	*N. tangutorum* wine: Meili rosé wine = 20%:80%
	NT‐ML‐4	*N. tangutorum* wine: Meili rosé wine = 25%:75%

“Shine Muscat” grape juice, highland barley wine blended with *N. tangutorum* wine	NT‐QK‐SM‐1	*N. tangutorum* wine: barley wine: “Shine Muscat” grape juice = 30%:30%:40%
	NT‐QK‐SM‐2	*N. tangutorum* wine: barley wine: “Shine Muscat” grape juice = 30%:35%:35%
	NT‐QK‐SM‐3	*N. tangutorum* wine: barley wine: “Shine Muscat” grape juice = 50%:30%:20%
	NT‐QK‐SM‐4	*N. tangutorum* wine: barley wine: “Shine Muscat” grape juice = 50%:25%:25%

### 2.3. Chemical Analysis of Blended Wine

Titratable acidity, reducing sugar, alcohol content, and volatile acidity (acetic acid, g•L^−1^) were measured in triplicate according to standard OIV methods [21]. Titratable acidity (tartaric acid, g•L^−1^) was determined by titration against 0.05 M sodium hydroxide to an endpoint of pH 8.2. Reducing sugar (glucose, g•L^−1^) was quantified by titration using Fehling’s reagent. Alcohol content (%v/v) was determined by distillation followed by density measurement. Color parameters were measured with a CM‐5 spectrophotometer (Konica Minolta, Inc., Japan) and expressed as L^∗^ (lightness), a^∗^ (red–green), and b^∗^ (yellow–blue) values. Chroma (C^∗^_ab_), and hue angle (h_ab_) were calculated as described by Ju et al. [22]. All measurements were performed in triplicate.

### 2.4. Determination of Phenolic Compounds

The total phenolic content of wine samples was quantified using the Folin–Ciocalteu assay, with results expressed as mg/L gallic acid equivalents. The total anthocyanin content was determined by the pH differential method [23], where the concentration was calculated from the absorbance difference at pH 1.0 and 4.5 and expressed as mg/L cyanidin‐3‐glucoside equivalents. The total flavonoid content was measured according to the NaNO_2_–AlCl_3_ spectrophotometric method, with results expressed as mg/L rutin equivalents. Total flavanol levels were determined using the p‐DMACA‐hydrochloric acid assay [24] and are reported as mg/L catechin equivalents. The tannin content was assessed by the methyl cellulose precipitation (MCP) method [25], with results also expressed as mg/L catechin equivalents.

### 2.5. Sensory Evaluation

A sensory panel of 20 evaluators (10 males and 10 females aged 20–30 years) with professional training and relevant educational backgrounds was assembled. Sensory evaluation was performed using a modified 100‐point method, adapted from the ISO standard and tailored to the characteristics of *N. tangutorum* berry [1]. Evaluation criteria were established based on previous studies and relevant literature (Table S2). The assessment was conducted in a controlled sensory room maintained at 20°C, in accordance with the Chinese National Standard GB/T 17,946‐2008. Panelists rated each blended wine sample using the established criteria. Between samples, water was provided for palate cleansing to minimize carryover effects. The most palatable wine, as determined by the overall evaluation results, was selected for the subsequent analysis.

### 2.6. GC‐MS Analysis

The volatile aroma components in wine and their concentrations were determined via GC‐MS according to the method described by Chen et al. [26]. The procedure was as follows: A 8‐mL wine sample was taken; 8 μL of 40 μg/L isooctyl alcohol internal standard solution and 2 g of sodium chloride were added, mixed, and sealed in a 20‐mL headspace bottle. Agilent 7890‐5975 GC‐MS was used for analysis. It is equipped with a DB‐WAX capillary gas chromatographic column (60 m × 250 μm × 0.25 μm, Agilent J & W, Santa Clara, CA), an Agilent 7683 automatic sampler (Agilent, Santa Clara, CA), and a solid phase microextraction head (50/30 μm, DVB/Carboxen/PDMS, Supelco, Bellefonte, PA, USA) for sample headspace solid phase microextraction (HS‐SPME). The carrier gas flow rate was 1 mL/min (He > 99.999%). The column oven temperature program was as follows: The initial temperature was 50°C, maintained for 1 min, and increased to 220°C at a rate of 3°C/min, maintained for 5 min. The temperature of the MS ion source is 230°C, the ionization mode is electron bombardment ionization, and the electron energy is 70 eV. The ion source, quadrupole, and transfer line temperatures were set at 230°C, 150°C, and 230°C, respectively. Each sample was analyzed in triplicate.

Identification and quantification. Volatile compounds were characterized by aligning their experimental retention indices (RIs) (calculated using a homologous series of n‐alkanes ranging from C7 to C24) and mass spectral data against those available from certified pure reference standards (Supelco, Bellefonte, PA, USA). All acquired mass spectra were further cross‐referenced with the National Institute of Standards and Technology Mass Spectral Library (NIST 11) to enhance identification confidence. For quantitative analysis, isooctyl alcohol was used as the internal standard. A five‐point calibration curve was prepared by the internal standard method, and target volatiles were quantified accordingly. Concentrations are expressed as mg/L of wine.

### 2.7. GC‐IMS Analysis

Analysis was performed using a FlavourSpec gas analysis system (G.A.S.) equipped with an MXT‐5 capillary column. For each run, 1 mL of liquid sample was placed into a 20‐mL headspace vial and incubated at 60°C for 10 min; subsequently, 100 μL of headspace gas was injected. Detailed instrumental parameters are listed in Table S3 and S4. Volatile compounds were identified by matching experimental RIs and drift times (DTs) against reference values from a homologous n‐ketone series (C4–C9) contained in the instrument’s built‐in IMS database, assisted by the Laboratory Analytical Viewer (LAV) software in conjunction with GC‐MS library matching. Data processing and visualization were carried out using the system’s integrated software package, which includes VOCal, Reporter, and Gallery Plot modules. VOCal enables spectral processing, qualitative/quantitative analysis, and compound identification via built‐in NIST and IMS databases, with the option to expand the database using user‐supplied standards. Quantitative results are obtained after establishing a calibration curve for each target compound. The Reporter plug‐in enables direct comparison of spectral differences between samples through 3D spectra, 2D top views, and differential spectra. The Gallery Plot plug‐in performs fingerprint comparisons, allowing visual and quantitative assessment of volatile organic compounds across different samples. Peaks corresponding to compounds not found in the migration spectral library are assigned numeric codes (Table S5).

### 2.8. Statistical Analysis

The data were analyzed by one‐way analysis of variance (ANOVA) followed by Duncan’s multiple range tests using IBM SPSS Statistics 21 (SPSS Inc., Chicago, USA). Differences at a significant level of 0.05 were considered significant. Plotting with GraphPad Prism 8.4.2. All measurements were performed in triplicate, and the results are presented as mean ± standard deviation. The heatmap was generated by using the pheatmap package of R (Version 4.3.3). PCA and PLS‐DA were performed using ade4 and mixOmics packages of R programming language (4.03), respectively.

## 3. Results and Discussion

### 3.1. Basic Physicochemical Characteristics of *N. tangutorum*–Blended Wine

Figure 1 shows the basic chemical composition of all wine samples prepared with different blending strategies. Acidity plays a crucial role in wine flavor, as it contributes directly to refreshing taste while lending vitality and balance to the wine [26, 27]. In this study, both titratable acidity and volatile acidity of the control (CK, *N. tangutorum* wine) were significantly higher than those of the other samples (*p* < 0.05; Figures 1(b), 1(c)). Among the blended wines, the *N. tangutorum*–barley–Shine Muscat blends (NT‐QK‐SM‐1–4) exhibited the lowest titratable acidity, with NT‐QK‐SM‐2 showing the minimum value (3.46 g/L). The highest volatile acidity recorded in the blended group was 0.68 g/L, while that of CK reached 1.17 g/L. The decrease in acidity may be attributed to interactions between organic acids and wine polyphenols, such as tannins and anthocyanins, which can form hydrogen bonds or undergo hydrophobic association, thereby reducing free acid concentration [28]. Shiraishi et al. [29] found that the higher the phenolic content in the four table grape varieties (Kyoho, Pione, Suzuka, Shine Muscat), the lower the titratable acid content. Similarly, Lu et al. [30] reported that mixed fermentation of black wolfberry and Cabernet Sauvignon led to a decrease in total acidity. Overall, blending *N. tangutorum*–Meili wine or *N. tangutorum*–barley–Shine Muscat wine effectively lowered both titratable and volatile acidity compared to *N. tangutorum* wine. Regarding reducing sugars, the *N. tangutorum*–barley–Shine Muscat blends contained significantly higher levels than CK (*p* < 0.05; Figure 1(a)). NT‐QK‐SM‐1 had the highest reducing‐sugar content, reaching 19.59 g/L, whereas the *N. tangutorum*‐Meili blends showed significantly lower reducing sugar than CK (*p* < 0.05). The elevated sugar in the *N. tangutorum*–barley–Shine Muscat blende likely arises from two factors: the inherently high reducing‐sugar content of the highland barley wine itself (QK‐SM showed the highest sugar level), and potential Maillard‐type interactions between sugars from barley wine and amino acids from *N. tangutorum* wine, during which degradation of Amadori intermediates may release further reducing sugars [31]. The alcohol content was significantly higher in all blended wines compared with CK (*p* < 0.05), with NT‐QK‐SM‐2 reaching 18.49% vol (Figure 1(d)). This increase may reflect a complementary enzyme activity between barley wine and *N. tangutorum* wine that improves saccharification efficiency, thereby boosting fermentable sugar availability and final ethanol yield [32]. In summary, blending *N. tangutorum* wine with Meili rose wine, or barley wine and Shine Muscat grape juice effectively reduced the naturally high acidity of the *N. tangutorum* wine while elevating the alcohol content. These adjustments diminish the need for exogenous acid adjustment or sugar supplementation during winemaking, thereby better preserving the intrinsic flavor and potential nutritional quality of the fruit wines.

FIGURE 1Basic physicochemical characteristics of *N. tangutorum* wine samples with different blending methods. Different letters represent the significant differences between the varieties according to ANOVA with Duncan’s test (*p* < 0.05). Reducing sugar content (a); titratable acidity content (b); volatile acidity content (c); alcohol content (d). See Table 1 for the wine samples.

**Figure:**
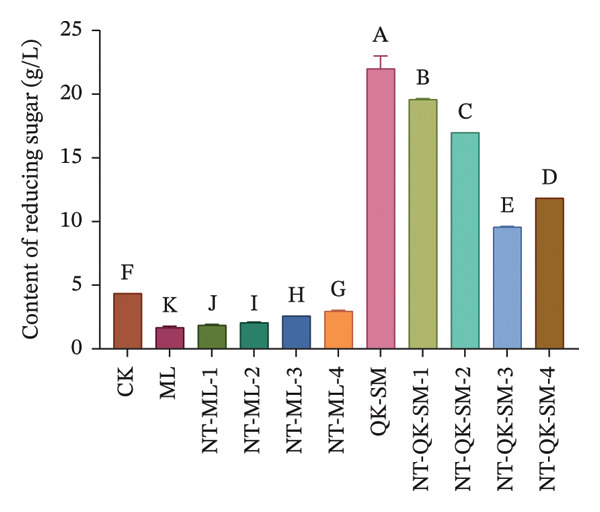
(a)

**Figure:**
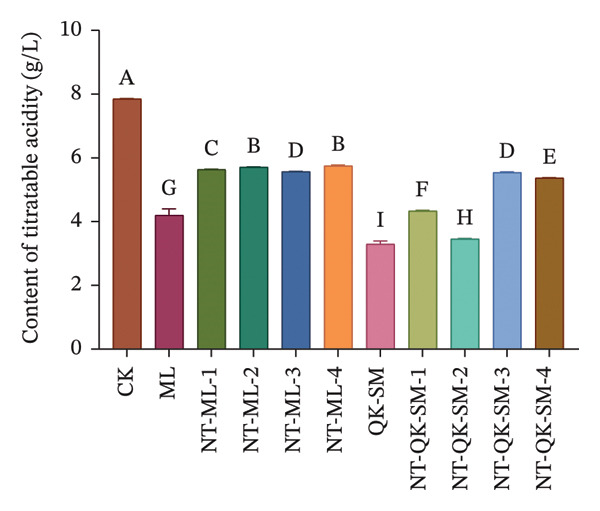
(b)

**Figure:**
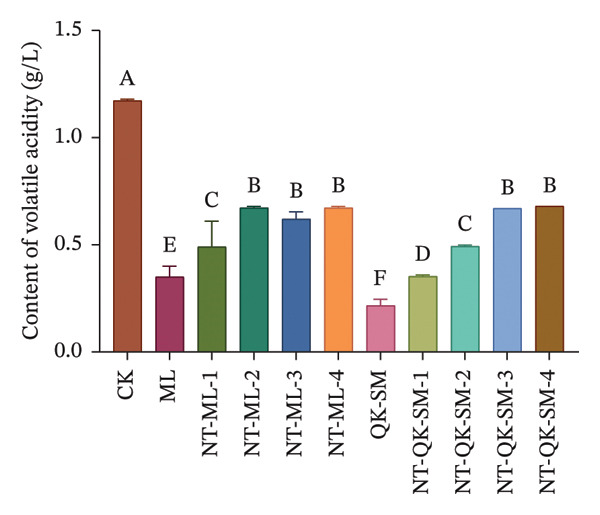
(c)

**Figure:**
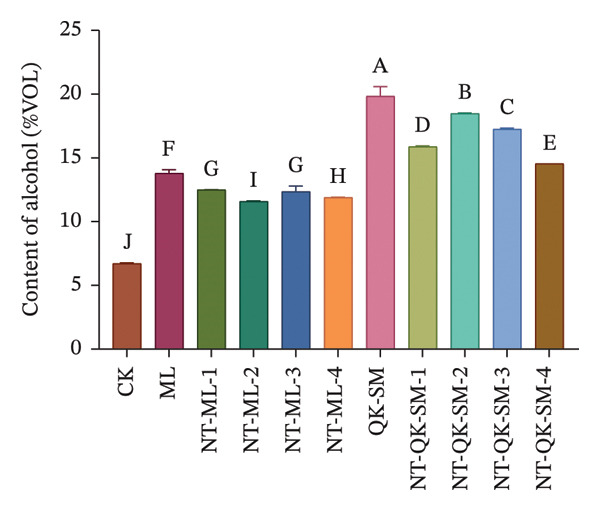
(d)

### 3.2. Phenolic and Color Parameters

Phenolic compounds are key determinants of wine color, astringency, and overall sensory structure. Wine polyphenols are commonly divided into flavonoids (e.g., anthocyanins and tannins) and nonflavonoids. Anthocyanins provide the initial color, while tannins contribute to astringency; both can further react to form stable polymeric pigments during aging [33]. As shown in Figure 2, CK exhibited higher levels of total phenols, flavonoids, and anthocyanins than all blended samples (*p* < 0.05; Figures 2(a), 2(b), 2(d)). This is consistent with previous reports that *N. tangutorum* is rich not only in amino acids, trace elements, and vitamin C but also in diverse phenolic compounds including flavonoids, phenolic acids, and alkaloids [34, 35], which explains the strong phenolic background of *N. tangutorum* wine. Interestingly, the *N. tangutorum* blended wines exhibited a distinct phenolic profile: Both flavanol and tannin contents were significantly elevated compared with CK. Specifically, NT‐ML‐2 showed the highest flavanol concentration (1.67 mg/L), and NT‐QK‐SM‐2 contained the most tannins (27.92 mg/L; Figure 2(e)). This increase primarily stems from the direct contribution of phenolics present in the added ingredients (Meili rose wine, barley wine, and Shine Muscat juice). Although the absolute phenolic content in the blends remained lower than in the pure additives, the rise relative to the base wine indicates effective retention of the introduced compounds. This retention may be facilitated by *N. tangutorum* components that stabilize phenolics through interactions such as copigmentation or complexation with proteins and polysaccharides, thereby limiting precipitation or degradation [36]. Tannins are critical for wine structure, contributing to mouthfeel, flavor complexity, and long‐term stability [37, 38]. In summary, although the *N. tangutorum* wine contained higher levels of certain phenolic classes, strategic blending successfully enhanced the concentration of flavanols and tannins. These compounds are key to the wine’s sensory structure and aging potential. The overall phenolic profile was thus improved through the complementary integration of phenolics from different sources.

FIGURE 2Phenolic content of *N. tangutorum* wine samples with different blending methods: Total phenol content; (a) flavonoid content (b); flavanol content (c); anthocyanin content (d); tannin content (e). Different letters represent the significant differences between the varieties according to ANOVA with Duncan’s test (*p* < 0.05). See Table 1 for the wine samples.

**Figure:**
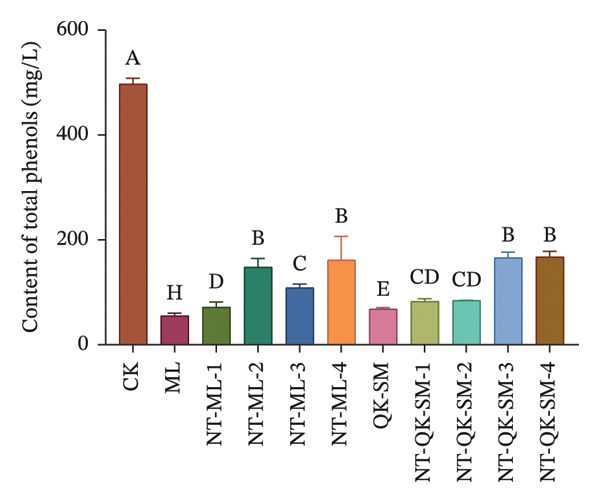
(a)

**Figure:**
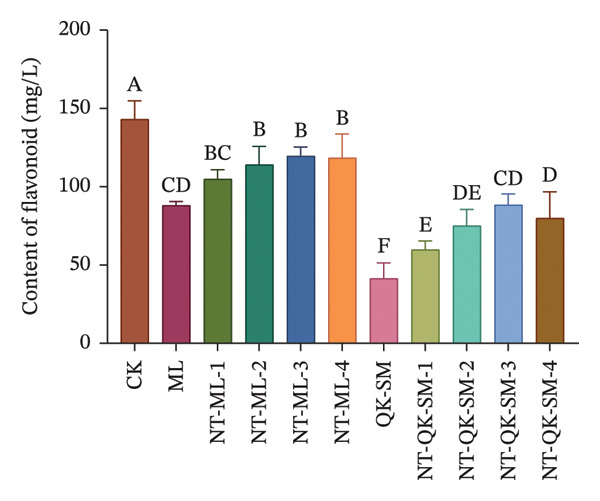
(b)

**Figure:**
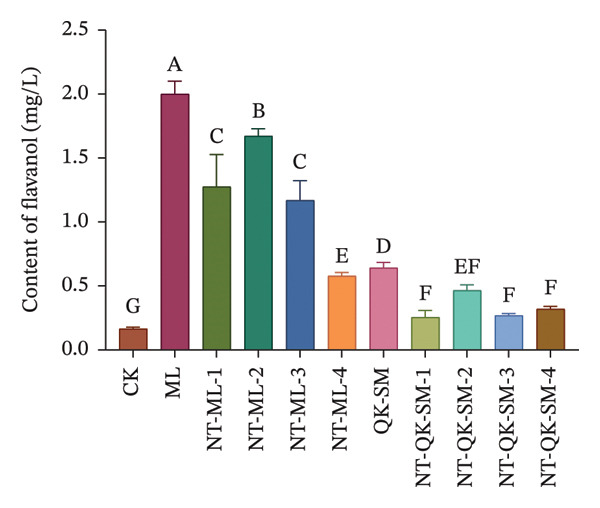
(c)

**Figure:**
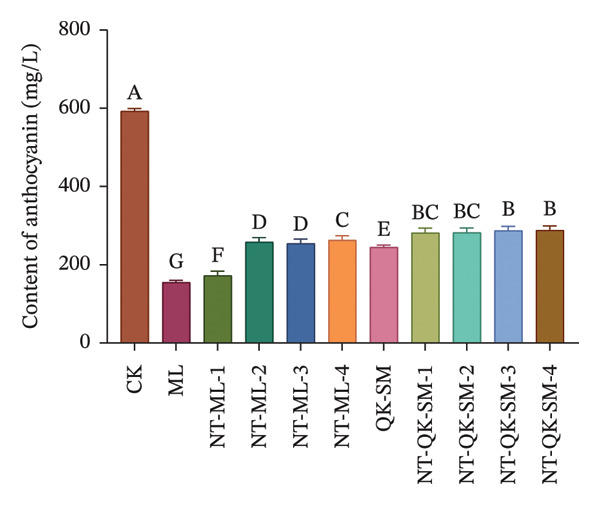
(d)

**Figure:**
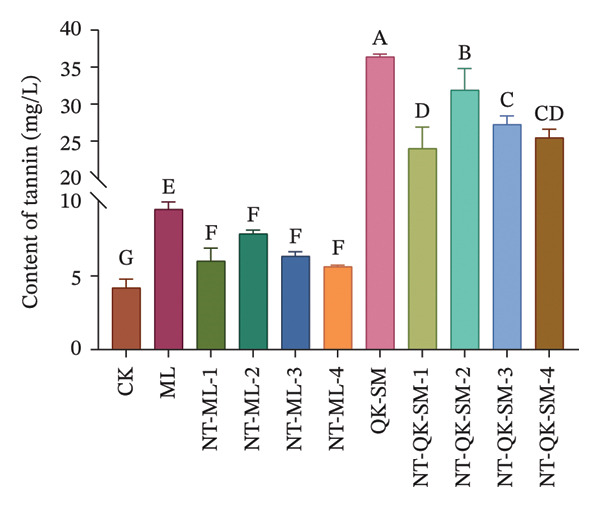
(e)

**FIGURE 3:**
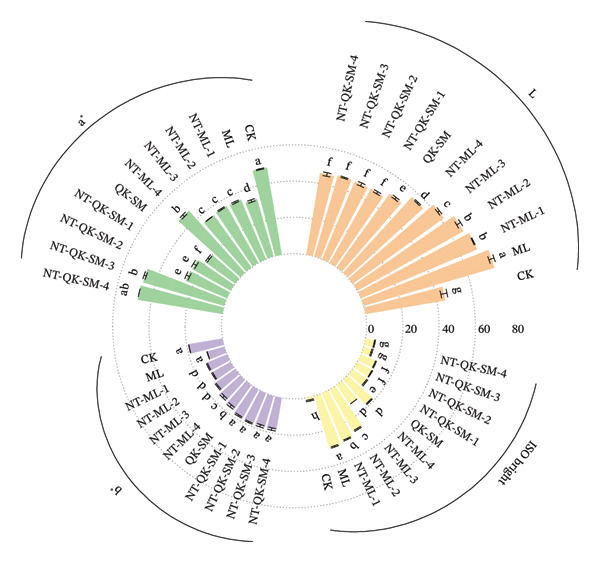
Color parameters of *N. tangutorum* wine samples with different blending methods. Different letters represent the significant differences between the varieties according to ANOVA with Duncan’s test (*p* < 0.05). See Table 1 for the wine samples.

Color analysis revealed that blending significantly altered chromatic parameters of the wines (Figure 3). Compared with CK, the blended wines showed significantly higher L^∗^ values (*p* < 0.05). Correspondingly, a^∗^ values were significantly lower in the blends (except NT‐QK‐SM‐4), and the b^∗^ values of NT‐ML blends were significantly lower than those of CK (*p* < 0.05). Notably, the ISO brightness of all blended wines was significantly higher than that of CK, indicating that blending effectively enhanced the luminosity of the *N. tangutorum* wine. The increase in brightness and the reduction in red tone likely result from interactions between the anthocyanins abundant in *N. tangutorum* wine and phenolic compounds introduced from the blending ingredients. Anthocyanins, the main pigments, can undergo copigmentation or form complexes with other phenolics (e.g., from Meili rose wine or Shine Muscat juice), which not only stabilizes color but also shifts hue and increases perceived brightness. This interaction thus contributes to a more stable and visually appealing wine color [39].

### 3.3. Sensory Analysis for Wine Selection

Quantitative Descriptive Sensory Analysis (QDA) was conducted to characterize the sensory profiles of the *N. tangutorum*–blended wines, with panelists rating the intensity of specific attributes [40]. The results are summarized in Table 2. The CK received a relatively low overall score, being described as purplish‐red with poor clarity, carrying off‐odors, and displaying slight astringency. In contrast, all blended wines scored higher than CK in appearance, aroma, and taste, although their style ratings were similar (*p* < 0.05). The NT‐QK‐SM‐2 achieved the highest scores across all sensory categories (appearance, aroma, taste, style, and overall evaluation). It was characterized as a clear, ruby‐red wine with a rich, harmonious, and refined aroma, a well‐defined style, and a balanced palate, performing significantly better than other samples. Based on these sensory results, NT‐ML‐2 and NT‐QK‐SM‐2 with the highest aroma scores were selected for the subsequent aroma components analysis to investigate the effect of different blending strategies on the aromatic profile of *N. tangutorum* wines.

**TABLE 2:** Sensory evaluation of *N. tangutorum*–blended wine.

	Appearance	Aroma	Taste	Style	Overall score
CK	8.95 ± 0.38^f^	16.25 ± 0.57^f^	24.8 ± 0.58^h^	8.5 ± 0.14^c^	58.5 ± 0.89^f^
ML	11.25 ± 0.16^d^	27.34 ± 0.23^e^	33.54 ± 0.25^cd^	9.1 ± 0.14^b^	81.23 ± 0.78^b^
QK‐SM	14.13 ± 0.22^a^	25.64 ± 0.43^b^	36.95 ± 0.82^b^	9.42 ± 0.11^a^	90.12 ± 0.25^a^
NT‐ML‐1	8.15 ± 0.15^g^	20.1 ± 0.44^e^	28.3 ± 0.65^g^	7.6 ± 0.15^d^	64.15 ± 0.99^e^
NT‐ML‐2	11.1 ± 0.27^d^	27.4 ± 0.38^a^	34.45 ± 0.37^cd^	9.5 ± 0.14^a^	82.45 ± 0.66^b^
NT‐ML‐3	10.25 ± 0.14^e^	23.85 ± 0.38^c^	33.25 ± 0.49^de^	8.4 ± 0.17^c^	75.75 ± 0.75^c^
NT‐ML‐4	10.55 ± 0.11^de^	19.2 ± 0.37^e^	29.9 ± 0.59^f^	7.65 ± 0.11^d^	67.3 ± 0.71^d^
NT‐QK‐SM‐1	12.5 ± 0.24^c^	25.65 ± 0.48^b^	36.65 ± 0.71^b^	8.8 ± 0.16^bc^	83.6 ± 1.21^b^
NT‐QK‐SM‐2	14.35 ± 0.17^a^	28.2 ± 0.24^a^	39.85 ± 0.33^a^	9.5 ± 0.14^a^	91.9 ± 0.38^a^
NT‐QK‐SM‐3	13.25 ± 0.20^b^	22.1 ± 0.51^d^	32.7 ± 0.65^e^	9.25 ± 0.16^a^	77.3 ± 1.06^c^
NT‐QK‐SM‐4	13.15 ± 0.17^b^	25 ± 0.46^bc^	35.05 ± 0.61^c^	9.15 ± 0.11^ab^	82.35 ± 0.62^b^

*Note:* Data are expressed as mean values of three independent experiments ± standard deviation (*n* = 20). Different letters represent the significant differences between the varieties according to ANOVA with Duncan’s test (*p* < 0.05). See Table 1 for the wine samples.

### 3.4. GC‐IMS Analysis of Volatile Profile in Wine With Different Blending Methods

GC‐IMS was employed for volatile profiling, a technique well established for a high‐resolution, rapid flavor analysis and effective isomer separation in food quality assessment [15, 41]. Using GC‐IMS, we effectively separated and visually distinguished the volatile components of the three wine samples. A recognized limitation of untargeted flavoromics using GC‐IMS is the frequent presence of unidentified signals, largely due to the lack of comprehensive commercial databases and the practical challenges of authentic standard verification. In this study, approximately 50% of the detected signals remained unassigned. Nevertheless, the complete spectral fingerprint—including these unknown features—successfully discriminated the samples, highlighting the utility of GC‐IMS for global flavor profiling. Future work will focus on expanding our in‐house library by integrating complementary techniques such as GC‐MS.

In Figure 4(a), each detected volatile compound is identified by a small dot to the right of the reactant ion peak (RIP). Using the CK sample as a reference, a difference plot was generated by subtracting the spectra of the other samples from that of CK (Figure 4(b)). In this plot, the white areas indicate the volatile concentrations similar to CK, the blue areas denote the lower concentrations, and the red areas signify the higher concentrations relative to CK. Based on all two‐dimensional spectral peaks, a unique volatile fingerprint for each blending treatment was constructed.

FIGURE 4GC‐IMS volatile compound analysis in *N. tangutorum* wine samples with different blending methods (*n* = 3). Two‐dimensional topographic plots of volatile compounds in CK, NT‐ML‐2, and NT‐QK‐SM‐2 samples. (a) NT‐ML‐2 and NT‐QK‐SM‐2 were reduced by using CK as a reference and the comparative analysis of topographic plots. (b) Fingerprints of volatile compounds in the three samples (CK, NT‐ML‐2, and NT‐QK‐SM‐2). (c) Principal component analysis (PCA) of three samples (CK, NT‐ML‐2, and NT‐QK‐SM‐2) (d).Note: in Figure 4(a), each detected volatile compound is identified by a small dot to the right of the RIP peak. Taking CK as the reference object, we subtracted the spectrum data of other samples from the signal peak of CK to generate the image after subtracting the reference (Figure 4(b)). Among them, white areas represent the aroma compounds with similar concentrations to the reference substance; blue areas indicate that the corresponding volatile component content in the sample is lower than CK; red areas indicate that these volatile components are present in higher amounts than CK in the sample. The numbers in the figure represent the undetermined substances in the migration spectrum library, which are replaced by numbers (Table S5).

**Figure:**
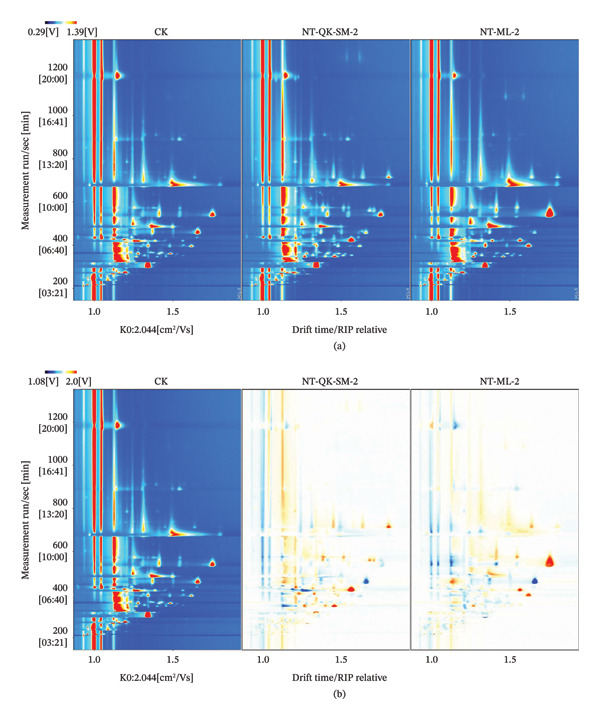

**Figure:**
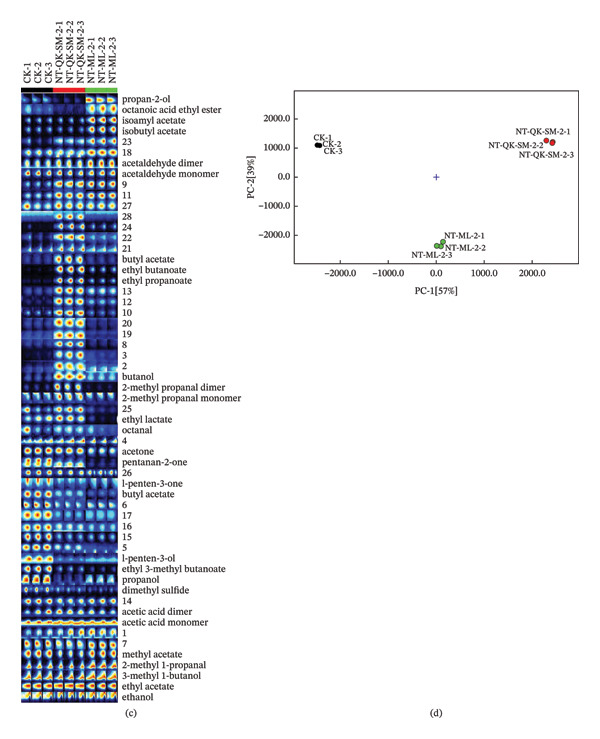

To visualize the compositional changes across samples, all detected peaks were extracted to construct a comparative characteristic fingerprint (Figure 4(c)). Although the three wine samples shared a largely similar set of volatile aroma compounds, the concentrations of individual substances varied considerably. The NT‐QK‐SM‐2 exhibited the highest overall content, most pronounced variations, and the greatest diversity of volatiles, followed by NT‐ML‐2. This outcome highlights the critical influence of blending ingredients (Meili rose wine, barley wine, and Shine Muscat grape juice) on the aroma profile of *N. tangutorum* wine. By selecting specific raw materials, winemakers can strategically modulate the volatile composition to enhance desired aroma notes and overall wine quality. For example, the elevated levels of certain esters and alcohols in NT‐QK‐SM‐2 likely contribute to more intense fruity and floral aromas, which are valued in premium *N. tangutorum* wines.

PCA was performed on multiple variables obtained from HS‐GC‐IMS to highlight the differences in volatile compound profiles. The variance contributions of PC1 and PC2 were 57% and 39%, respectively, with a cumulative variance of 96%, significantly higher than the acceptable threshold (Figure 4(d)). The PCA results revealed significant differences in aroma compounds among the three sample groups, effectively distinguishing them from each other. Notably, NT‐ML‐2 had larger concentrations of acetaldehyde, isobutyl acetate, isoamyl acetate, ethyl caprylate, and 2‐propyl alcohol compared to the other wines. In contrast, NT‐QK‐SM‐2 had larger amounts of ethyl lactate, 2‐methyl propanol, butanol, ethyl propionate, ethyl butyrate, and butyl acetate than the other wines. These compounds are known for their distinctive aromatic profiles, including honey, fruit, and floral notes, which significantly shape the sensory perception of wine.

In summary, HS‐GC‐IMS fingerprinting demonstrates that blending significantly modifies the concentration and composition of volatile compounds in *N. tangutorum* wine. The technique effectively separates and visualizes these differences across samples, confirming its practical value for both research and quality control in this wine system.

### 3.5. GC‐MS Analysis of Volatile Profile of *N. tangutorum*–Blended Wine

Aroma is a key sensory attribute of wine, shaped by factors such as grape variety, climate, fermentation conditions, and winemaking practices [42, 43] and plays a decisive role in defining wine typicity [44]. In the present study, the GC‐MS analysis of the *N. tangutorum*–blended wines identified 65 volatile compounds. As summarized in Table 3, these compounds were categorized into esters (21), higher alcohols (19), terpenes (9), fatty acids (6), aldehydes (5), phenols (3), and other aromatics (2).

**TABLE 3:** Volatile compounds (mg/L) of in *N. tangutorum*–blended wines with different blending methods.

Compound	RI	Thresholds (mg/L)	CK	ML	QK‐SM	NT‐ML‐2	NT‐QK‐SM‐2
*Esters*							
(±)‐5‐Butyl‐4‐methyldihydro‐2 (3H)‐furanone	2367		0.007 ± 0.001	0.004 ± 0	ND	0.005 ± 0	ND
β‐Phenethyl acetate	1820	0.21	0.062 ± 0.002^c^	0.145 ± 0.01^a^	0.062 ± 0.004^c^	0.125 ± 0.007^b^	0.063 ± 0.001^c^
Hexyl acetate	1289	0.67	0.362 ± 0.003^a^	0.205 ± 0.012^c^	0.644 ± 0.091^e^	0.297 ± 0.017^b^	0.109 ± 0.004^d^
Ethyl succinate	1328	200	0.55 ± 0.009^e^	0.832 ± 0.002^c^	1.322 ± 0.094^a^	0.714 ± 0.006^d^	1.124 ± 0.052^b^
Ethyl 2‐methylbutanoate	1056	0.018	0.006 ± 0	ND	0.005 ± 0	0.004 ± 0	0.011 ± 0.004
Ethyl 3‐hydroxybutyrate	1300		0.067 ± 0^a^	ND	0.021 ± 0^c^	0.022 ± 0^c^	0.036 ± 0.002^b^
Ethyl 2‐hydroxybenzoate	2598	15.781	1.92 ± 0.001^a^	1.743 ± 0.002^c^	1.745 ± 0.012^c^	1.89 ± 0.048^ab^	1.806 ± 0.001^b^
Ethyl 3‐methylbutanoate	1068	0.003	0.033 ± 0^a^	0.020 ± 0.007^b^	0.030 ± 0.005^ab^	0.013 ± 0^c^	0.031 ± 0.008^ab^
Ethyl dodecanoate	1640	1.5	0.044 ± 0.001^c^	0.061 ± 0.003^a^	0.023 ± 0.006^d^	0.051 ± 0^b^	0.043 ± 0^c^
Ethyl acetate	887	7.5	120.672 ± 4.972^e^	854.411 ± 4.101^a^	632.141 ± 3.231^c^	768.723 ± 3.922^b^	535.007 ± 47.540^d^
Ethyl nonanoate	1520	1.3	0.01 ± 0^c^	0.021 ± 0.003^a^	ND	0.013 ± 0.001^b^	0.011 ± 0^bc^
Ethyl caprylate	1888		0.272 ± 0.003^e^	0.954 ± 0.078^a^	0.432 ± 0.021^c^	0.847 ± 0.066^b^	0.399 ± 0.011^d^
Ethyl caproate	1577	0.014	0.14 ± 0.001^e^	0.732 ± 0.021^c^	1.342 ± 0.154^a^	0.614 ± 0.019^d^	1.085 ± 0.126^b^
Isobutyl acetate	1001	1.6	0.112 ± 0.003^c^	0.278 ± 0.011^a^	ND	0.222 ± 0.009^b^	0.053 ± 0.008^d^
Isopentyl hexanoate	1749	0.05	0.005 ± 0^d^	0.008 ± 0^b^	0.009 ± 0^a^	0.006 ± 0^c^	0.006 ± 0^c^
Methyl salicylate	1583	0.04	0.009 ± 0	ND	ND	0.009 ± 0	0.009 ± 0
Methyl caprylate		0.005	0.008 ± 0^c^	0.011 ± 0.001^a^	0.006 ± 0^e^	0.009 ± 0^b^	0.007 ± 0^d^
Ethyl lactate	1206	157.81	28.323 ± 1.576^c^	10.432 ± 0.547^e^	100.356 ± 6.783^a^	14.729 ± 1.047^d^	85.242 ± 5.291^b^
Ethyl isobutyrate	1240		0.316 ± 0.015^e^	0.765 ± 0.054^c^	1.354 ± 0.048^a^	0.595 ± 0.039^d^	1.185 ± 0.071^b^
Ethyl butyrate	1187	0.035	0.024 ± 0.001^e^	0.324 ± 0.007^c^	1.564 ± 0.142^a^	0.206 ± 0.008^d^	1.382 ± 0.275^b^
Isoamyl acetate	1134	0.03	0.468 ± 0.01^c^	5.634 ± 0.243^a^	0.134 ± 0.053^e^	4.163 ± 0.178^b^	0.268 ± 0.05^d^
Subtotal			151.180 ± 6.581^e^	876.581 ± 4.543^a^	741.192 ± 5.645^c^	793.265 ± 5.306^b^	625.988 ± 42.608^d^

*Alcohols*							
1‐Butanol	1109	150	1.313 ± 0.051^e^	3.975 ± 0.285^c^	7.564 ± 0.365^a^	2.272 ± 0.109^d^	5.140 ± 0.218^b^
3‐Methyl‐1‐butanol	1166	30	52.604 ± 2.5691^e^	254.385 ± 3.543^a^	78.453 ± 3.965^c^	209.797 ± 13,489.046^b^	68.396 ± 4.181^d^
4‐Methyl‐1‐pentanol	1345	50	49.093 ± 1.335^a^	20.545 ± 0.954^c^	6.987 ± 0.564^e^	32.629 ± 1.606^b^	7.836 ± 0.474^d^
1‐Decanol	1783	0.4	0.143 ± 0^b^	0.141 ± 0^c^	0.145 ± 0^a^	0.145 ± 0^a^	0.143 ± 0^b^
1‐Heptanol	1313	2.5	0.025 ± 0.001^e^	2.192 ± 0.154^a^	0.934 ± 0.027^c^	1.730 ± 0.186^b^	0.471 ± 0.031^d^
1‐Hexanol	1336	8	0.218 ± 0.022^e^	0.315 ± 0.019^c^	0.425 ± 0.056^a^	0.272 ± 0.02^d^	0.394 ± 0.045^b^
2‐Ethyl‐1‐hexanol	1451	8	0.012 ± 0.001^a^	ND	ND	0.007 ± 0^b^	0.007 ± 0^b^
1‐Octanol	1627	0.04	0.021 ± 0^e^	0.054 ± 0.002^a^	0.029 ± 0.001^c^	0.035 ± 0.001^b^	0.025 ± 0.001^d^
1‐Octen‐3‐ol	1382	0.001	0.014 ± 0^e^	0.075 ± 0.003^a^	0.044 ± 0.002^c^	0.053 ± 0.004^b^	0.025 ± 0.001^d^
1‐Pentanol	1267	1	ND	10.432 ± 0.155^b^	18.564 ± 1.365^a^	5.697 ± 0.233^c^	11.060 ± 1.306^b^
1‐Propanol	996	306	52.336 ± 2.164^e^	60.354 ± 3.732^c^	80.291 ± 4.093^a^	59.924 ± 3.602^d^	72.768 ± 2.391^b^
2‐Methyl‐1‐propanol	1050	40	27.065 ± 1.244^e^	162.543 ± 7.786^a^	54.981 ± 1.657^c^	101.692 ± 6.722^b^	34.043 ± 1.301^d^
3‐Methylthiopropanol	1727	0.5	0.352 ± 0.004^d^	6.753 ± 0.354^a^	0.546 ± 0.035^c^	3.500 ± 0.219^b^	0.365 ± 0.059^d^
(*E*)‐2‐Hexen‐1‐ol	1364	0.4	0.042 ± 0.004^a^	0.006 ± 0.001^d^	0.011 ± 0.001^c^	0.011 ± 0.001^c^	0.018 ± 0.001^b^
(*Z*)‐2‐Hexen‐1‐ol	1369	0.4	0.644 ± 0.571^e^	31.564 ± 1.054^a^	9.362 ± 0.798^c^	20.157 ± 2.081^b^	5.727 ± 0.404^d^
(*E*) ‐3‐Hexen‐1‐ol	1346	0.4	0.021 ± 0.002^e^	0.102 ± 0.004^c^	1.465 ± 0.054^a^	0.072 ± 0.002^d^	0.611 ± 0.006^b^
(*Z*) ‐3‐Hexen‐1‐ol	1351	0.4	0.226 ± 0.018^a^	ND	ND	0.032 ± 0.001^b^	0.059 ± 0.004^b^
Benzyl alcohol	1538	200	1.659 ± 0^a^	0.522 ± 0.066^c^	0.776 ± 0.02^b^	0.522 ± 0.066^c^	0.776 ± 0.02^b^
Phenylethyl alcohol	1532	10	12.799 ± 0.06^b^	25.193 ± 0.922^a^	10.925 ± 0.378^b^	25.193 ± 0.922^a^	10.925 ± 0.378^b^
Subtotal			149.549 ± 5.5714^e^	579.151 ± 8.475^a^	271.502 ± 4.829^c^	431.150 ± 27.535^b^	210.965 ± 6.043^d^

*Fatty acids*							
Benzoic acid	1651		0.182 ± 0.001	0.201 ± 0.034	0.197 ± 0.009	0.189 ± 0.007	0.192 ± 0.015
Isopentanoic acid	1568	0.033	0.451 ± 0.02^a^	ND	0.107 ± 0.007^c^	0.251 ± 0.004^b^	0.237 ± 0.005^b^
Hexanoic acid	1843	3	3.125 ± 0.184^a^	0.991 ± 0.032^e^	1.021 ± 0.023^d^	1.275 ± 0.044^b^	1.157 ± 0.048^c^
n‐Decanoic acid	2483	1	0.127 ± 0.001^c^	0.184 ± 0.006^a^	0.113 ± 0.004^d^	0.164 ± 0.005^b^	0.126 ± 0.007^c^
Isobutyric acid	1620	2.3	0.284 ± 0.001	0.267 ± 0.002	0.283 ± 0.008	0.257 ± 0.001	0.293 ± 0.005
Octanoic acid	2030	0.5	0.442 ± 0.001^b^	0.894 ± 0.031^a^	0.433 ± 0.006^b^	0.864 ± 0.025^a^	0.453 ± 0.008^b^
Subtotal			4.612 ± 0.205^a^	2.537 ± 0.055^c^	2.154 ± 0.012^e^	3.037 ± 0.095^b^	2.431 ± 0.041^d^

*Aldehydes*							
Benzaldehyde	1517		0.162 ± 0.001^a^	0.012 ± 0.001^c^	0.132 ± 0.018^b^	0.049 ± 0.006^c^	0.154 ± 0.018^a^
Decanal	1710	0.001	0.001 ± 0	ND	ND	0.001 ± 0	0.001 ± 0
Furfural	1459	14.1	0.082 ± 0^d^	0.164 ± 0.017^c^	0.567 ± 0.001^a^	0.101 ± 0.017^d^	0.451 ± 0.001^b^
Nonanal	1672	0.001	0.013 ± 0^c^	0.012 ± 0^d^	0.015 ± 0^a^	0.013 ± 0^c^	0.014 ± 0^b^
Octanal	1454		0.107 ± 0.003^a^	0.049 ± 0^c^	ND	0.079 ± 0^b^	0.006 ± 0.001^d^
Subtotal			0.364 ± 0.002^c^	0.237 ± 0.001^d^	0.714 ± 0.008^a^	0.224 ± 0.012^e^	0.626 ± 0.004^b^

*Terpenes*							
Farnesol	2456	0.02	0.045 ± 0^c^	0.043 ± 0^e^	0.044 ± 0^d^	0.046 ± 0^b^	0.047 ± 0^a^
Nerol	2072		0.012 ± 0^a^	0.003 ± 0^c^	0.002 ± 0^c^	0.007 ± 0^b^	0.007 ± 0^b^
α‐Terpineol	1682	0.033	0.025 ± 0^a^	0.005 ± 0^d^	ND	0.012 ± 0^b^	0.007 ± 0^c^
Citronellol	1734	0.01	0.011 ± 0^b^	0.010 ± 0^c^	0.013 ± 0^a^	0.011 ± 0^ab^	0.011 ± 0^a^
Geraniol	1575	0.03	0.026 ± 0^a^	0.010 ± 0^b^	0.009 ± 0^b^	0.015 ± 0^c^	0.015 ± 0^c^
Geranyl acetate	2057		0.008 ± 0	ND	ND	0.008 ± 0	0.008 ± 0
Linalool	1602	0.025	0.018 ± 0^b^	0.016 ± 0.003^d^	0.020 ± 0.001^a^	0.017 ± 0^c^	0.016 ± 0.001^d^
D‐Limonene	1285		0.013 ± 0.001^c^	0.014 ± 0.002^c^	0.023 ± 0.004^a^	0.013 ± 0.001^c^	0.017 ± 0^b^
(+)‐rose oxide	1652		0.007 ± 0^a^	0.003 ± 0^b^	0.002 ± 0^b^	0.004 ± 0^b^	0.004 ± 0^b^
Subtotal			0.137 ± 0.025	0.104 ± 0.021	0.113 ± 0.011	0.129 ± 0.015	0.124 ± 0.021

*Phenols*							
Phenol	2073		0.036 ± 0.001	0.034 ± 0	0.033 ± 0	0.038 ± 0	0.038 ± 0
4‐Ethylphenol	2304	0.44	0.063 ± 0.001^a^	0.014 ± 0.001^c^	0.013 ± 0^c^	0.026 ± 0.002^b^	0.024 ± 0^b^
2‐Methyl‐4‐ethylphenol	2130		0.035 ± 0	ND	0.034 ± 0	0.035 ± 0	ND
Subtotal			0.135 ± 0.001^a^	0.048 ± 0.001^e^	0.081 ± 0.002^c^	0.099 ± 0.002^b^	0.061 ± 0^d^

*Other*							
β‐Ionone	1932	0.1	0.004 ± 0	0.005 ± 0	ND	0.004 ± 0	ND
2‐Isobutyl‐3‐methoxypyrazine	1525		0.008 ± 0	0.007 ± 0	ND	0.008 ± 0	ND
Total			306.052 ± 2.218^e^	1458.669 ± 5.463^a^	1015.753 ± 3.546^b^	533.975 ± 2.343^d^	855.141 ± 3.786^c^

*Note:* All parameters are listed with their standard deviations (*n* = 3) (mg/L). For each parameter, values with different letters are significantly different between the samples (*p* ≤ 0.05).

Abbreviations: ND, not detected; RI, retention indices.

Esters, which impart the characteristic fruity notes of wine, are key contributors to its aroma profile [42]. Their production depends on multiple winemaking factors, including temperature, yeast strain, and aeration [45]. In this study, 21 esters were detected. The total ester content in NT‐ML‐2 (793.27 mg/L) and NT‐QK‐SM‐2 (625.99 mg/L) was significantly higher than in CK (*p* < 0.05), although the Meili rosé wine (ML) itself contained the highest level (876.58 mg/L). Ethyl acetate was the most abundant ester. Its concentration in NT‐ML‐2 and NT‐QK‐SM‐2 was 6.37 and 4.43 times that in CK, respectively. The high ethyl acetate content in NT‐ML‐2 is attributed primarily to the inherent richness of this compound in Meili rosé wine [46]. The blending process itself further modulates ester formation, as reported in mixed fermentations of barley wort and grape juice, where ethyl acetate and isovalerate levels are strongly influenced by the blending ratio [47]. Similarly, ethyl acetate content increased by 11.2% in a pear–grape–mixed beverage [8]. Other esters, such as ethyl 3‐hydroxybutyrate and ethyl caproate, were present at lower concentrations (0.03–1.75 mg/L). Despite their low levels, these compounds significantly enhance fruity and floral aromas due to their low olfactory thresholds [48, 49].

Higher alcohols are primarily formed via amino acid decarboxylation and deamination or through sugar metabolism during fermentation [50]. In the present study, the average content of higher alcohols in the *N. tangutorum*–blended wines ranged from 210.97 to 431.15 mg/L, which was 1.41–2.88 times higher than in CK (*p* < 0.05). Among the 19 higher alcohols detected, 3‐methyl‐1‐butanol was the most abundant, with an average content of 132.73 mg/L—a result consistent with reports on blended barley malt wort and grape wines [47]. Many higher alcohols contribute pleasant aromatic notes. For example, phenylethyl alcohol imparts aromas of honey and rose [51], and its concentration has been shown to increase in blended grape–pear juice, enhancing fruity and floral aromas [8]. In contrast, although 4‐methyl‐1‐pentanol and 1‐propanol were present at average levels of 23.42 and 65.13 mg/L, respectively, their concentrations in all samples remained below their reported odor thresholds, indicating a limited direct impact on the overall aroma.

Fatty acids in wine are formed from must composition and fermentation conditions. They are typically associated with fruity, cheesy, or rancid notes [52]. The average fatty acid content was highest in CK (4.61 mg/L), followed by NT‐ML‐2 (3.04 mg/L) and NT‐QK‐SM‐2 (2.43 mg/L). Among the six fatty acids detected, octanoic acid and n‐decanoic acid were present at higher levels in NT‐ML‐2 (0.86 and 1.28 mg/L, respectively) compared to CK (*p* < 0.05). Even at subthreshold concentrations, volatile fatty acids such as octanoic acid can contribute to aroma complexity [53].

Aldehydes are intermediate metabolites in wine fermentation, formed from corresponding keto acids derived from sugar or amino acid metabolism, and are subsequently reduced to their corresponding alcohols [54]. In the *N. tangutorum*–blended wines, NT‐QK‐SM‐2 showed the highest furfural content (0.45 mg/L), significantly higher than that of CK (*p* < 0.05). Nonanal levels in both NT‐ML‐2 and NT‐QK‐SM‐2 exceeded their odor threshold (0.005 mg/L), contributing spicy and tropical fruit notes [55]. Although terpene concentrations were generally low in the wines studied, their distinct floral character often plays a key role in shaping the aroma profile [56]. No significant differences in the total terpenoid content were observed among samples; however, farnesol and D‐limonene levels in NT‐QK‐SM‐2 (0.05 and 0.02 mg/L, respectively) were significantly higher than in CK (*p* < 0.05). In terms of total aroma, the NT‐QK‐SM‐2 and NT‐ML‐2 were significantly higher than CK (*p* < 0.05), with NT‐ML‐2 reaching 855.14 mg/L. These results demonstrate that blending effectively enhances the overall aroma intensity of *N. tangutorum* wine.

### 3.6. Odor Activity Value (OAV)–Based Characteristic Aroma Analysis

Although numerous volatile compounds were detected in the wines, not all significantly influence the overall aroma [57]. To assess the sensory contribution of individual volatiles, OAVs were calculated by dividing the average concentration of each compound by its published odor threshold [26, 57]. Compounds with OAV ≥ 1 are generally considered to contribute to the aroma (Tables S6 and S7). In this study, 21 compounds exhibited OAVs greater than 1, suggesting their potential role in shaping the aromatic profile of the wines.

However, among the eight compounds analyzed, OAVs exceeding 1 were observed only in certain samples. For example, ethyl butyrate, a C6 compound exhibiting typical characteristics of tart fruit, strawberry aromas [58], had an OAV of > 1 in all samples except CK. This compound is considered a key aroma marker that distinguishes barley wine from light‐aroma baijiu, typically showing higher concentration in the former [59], which aligns with our results: ethyl butyrate exhibited the highest OAV in the QK‐SM base wine, and its OAV in NT‐QK‐SM‐2 was significantly higher than in CK. Similarly, 1‐pentanol, which contributes fruity and sweet characteristics [53], showed OAVs > 1 in NT‐ML‐2 and NT‐QK‐SM‐2, but not in CK. Furfural, a primary contributor to pungent aroma [60], had an OAV exceeding 1 only in NT‐QK‐SM‐2. Its enhanced aromatic impact in this blend may be due to matrix effects—such as ethanol content, acidity, and sugar levels—that influence its volatility or perception.

The remaining 13 compounds with OAVs exceeding one comprised five esters, four alcohols, two terpenes, one fatty acid, and one aldehyde. Among the esters, ethyl acetate and ethyl caproate showed the highest OAVs (10.09–125.31). Ethyl acetate reached its maximum in NT‐ML‐2, followed by the ML base wine; ethyl caproate peaked in NT‐QK‐SM‐2, with QK‐SM ranking second. Both compounds contribute sweet, fruity notes that can enhance the wine’s overall flavor complexity [61, 62]. Higher alcohols generally exert a positive influence on wine aroma when their total concentration remains below 400 mg/L [63]. 1‐Octen‐3‐ol, a straight‐chain fatty alcohol and the primary contributor to mushroom aroma [51], exhibited higher OAVs in NT‐QK‐SM‐2 and NT‐ML‐2 than in CK (*p* < 0.05). The elevated OAVs in these blends reflect the substantial amounts present in the ML and QK‐SM base wines, indicating that blending effectively raised the level of this character‐impact compound. By contrast, the OAV of isopentanoic acid was significantly lower in the blended wines than in CK (*p* < 0.05). Although volatile fatty acids can add complexity at subthreshold concentrations, exceeding their perception threshold can impart unpleasant notes. The blending process therefore helped reduce the potential negative aroma contribution from this fatty acid.

These results demonstrate that blending significantly modifies the aromatic profile of *N. tangutorum* wine. Clarifying the contribution patterns of key aroma compounds can guide the process design of blended wines, enabling the targeted optimization of the desired aroma characteristics. Future studies should focus on elucidating the underlying biochemical pathways, such as microbial metabolism and enzymatic reactions, that are involved in the formation of key aroma compounds during blending. This will provide a theoretical foundation for rational raw material selection and process control, ultimately allowing for the precise modulation of the aromatic quality in blended wines.

### 3.7. PCA and Partial Least Squares Discriminant Analysis (PLS‐DA)

PCA was performed to evaluate the influence of blending methods on the wine aroma profile, using 65 volatile compounds. As shown in Figure 5(a), using PCA‐X to visualize the relationship between samples, PC1 and PC2 accounted for 76.1% of the total variance (36.6% and 39.5%, respectively). The loading plot revealed positive correlations between PC1 and compounds such as 1‐pentanol, ethyl caproate, 1‐propanol, ethyl isobutyrate, 1‐hexanol, ethyl succinate, and 1‐butanol, suggesting that PC1 is primarily influenced by esters and alcohols (Figure 5(b)). These compounds are abundant in the NT‐QK‐SM‐2 and contribute violet, clove‐like floral, and grass aromas [51]. PC2 showed positive correlations with octanoic acid, 3‐methylthiopropanol, and 2‐methyl‐1‐propanol, and negative correlations with benzaldehyde, isobutyric acid, and benzyl alcohol. Positively correlated compounds like octanoic acid and 2‐methyl‐1‐propanol are known to contribute to fruity and floral aromas, while negatively correlated compounds such as benzaldehyde impart almond‐like notes, affecting the overall sensory profile of the wine [51, 64].

FIGURE 5PCA and PLS‐DA analysis of volatile profile in *N. tangutorum* wine samples with different blending methods. PCA scores (a); PCA loadings (b); VIP values from PLS‐DA (c).

**Figure:**
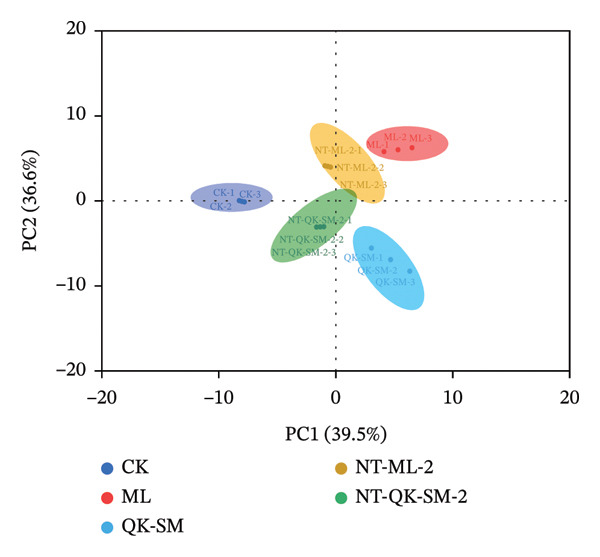
(a)

**Figure:**
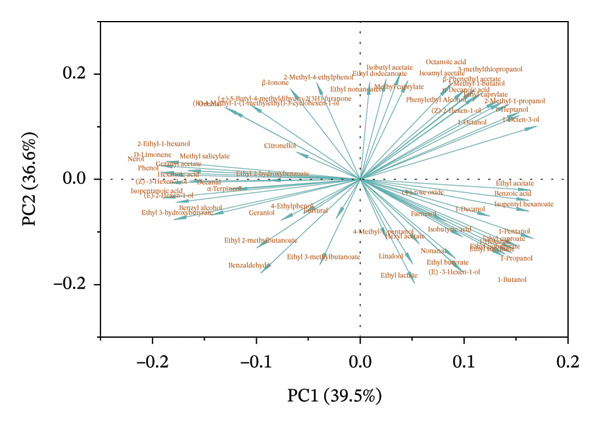
(b)

**Figure:**
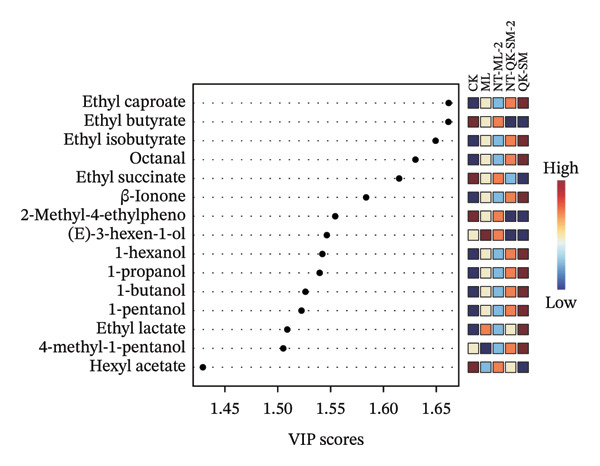
(c)

To further discriminate the aroma characteristics, PLS‐DA was performed based on the results of PCA. Variable importance in projection (VIP) scores was used to identify the most discriminative compounds (Figure 5(c)), with compounds scoring greater than 1 considered key contributors. Ten compounds were identified as major discriminators: ethyl caproate, ethyl butyrate, ethyl isobutyrate, octanal, ethyl succinate, β‐ionone, 2‐methyl‐4‐ethylphenol, (E)‐3‐hexen‐1‐ol, 1‐hexanol, and 1‐propanol. These volatile compounds are essential for assessing aroma differences among the *N. tangutorum*–blended wines. Identifying such key metabolites holds practical value for industrial production, as it can guide process adjustments to selectively enhance or attenuate specific aromas, thereby enabling precise flavor design of *N. tangutorum*–blended wines.

## 4. Conclusion

The blending technique enhanced the overall quality of *N. tangutorum* wine. The addition of Meili rose wine, barley wine, and Shine Muscat grape juice effectively lowered both titratable and volatile acidity while increasing flavanol and tannin levels and improving color brightness. Among various blending combinations, NT‐QK‐SM‐2 (30%:35%:35%) demonstrated particularly outstanding comprehensive performance. It exhibited higher reducing‐sugar and alcohol contents, resulting in a sweeter and smoother palate, while also achieving the highest concentrations of key aromatic compounds such as ethyl acetate, ethyl hexanoate, and ethyl butyrate. In contrast, NT‐ML‐2 (15%:85%) exhibited higher concentrations of flavor compounds such as methyl caproate, phenethyl alcohol, and citronellol. VIP scoring analysis identified ethyl caproate, ethyl butyrate, ethyl isobutyrate, octanal, ethyl succinate, β‐ionone, 2‐methyl‐4‐ethylphenol, (E)‐3‐hexen‐1‐ol, 1‐hexanol, and 1‐propanol as the ten compounds contributing most significantly to the aroma profile. In summary, blending technology effectively enhances the sensory quality of *N. tangutorum* wine by optimizing physicochemical properties and increasing aromatic complexity, providing a foundation for developing innovative, high‐quality fermented beverages.

## Author Contributions

Hao Chen: conceptualization, methodology, formal analysis, investigation, resources, data curation, writing–original draft, and writing–review and editing. Ying Li: methodology, formal analysis, investigation, data curation, writing–original draft, and writing–review and editing. Ming Chi: methodology, formal analysis, data curation, and writing–review and editing. Yuting Wang: methodology and writing–review and editing. Numan Khan: methodology, resources, and supervision. Ni Yang: methodology and resources. Xuefei Wang: formal analysis, investigation, and writing–review and editing. Yanzhen Zhang: methodology and resources. Fei Wang: writing–review and editing. Yuhua Bao: writing–review and editing. Yulin Zhang: resources, writing–review and editing, supervision, project administration, and funding acquisition. Zhumei Xi: conceptualization, resources, writing–review and editing, supervision, project administration, and funding acquisition.

## Funding

This study was supported by the Ningxia Hui Autonomous Region Key R&D project (Grant No. 2023BCF01023, the Key Research and Transformation Program Project of Qinghai Province (Grant No. 2024‐SF‐C06), and the Shaanxi Province Key R & D Project (Grant No. 2024NC‐YBXM‐019).

## Ethics Statement

This article does not contain any studies with animal subjects. All procedures performed in the process of *Nitraria tangutorum* Bobrov.–blended wines tasting involving human participants were in accordance with the ethical standards of the institutional. All sensory evaluation procedures are conducted in accordance with applicable ethical standards, and participants provided informed consent prior to testing. All tasters participated voluntarily in this experiment, adhering to the principles of the 1975 Declaration of Helsinki. Ethical approval for the sensory evaluation in this study was obtained from the Northwest A&F University Research Ethics Committee, with Reference No. NWAFUHR‐20230812.

## Conflicts of Interest

The authors declare no conflicts of interest.

## Supporting Information

Supporting Table S1: Soluble solids and titratable acid contents in the first three raw materials prior to fermentation.

Supporting Table S2: Overall sensory evaluation criteria of N. tangutorum–blended wine.

Supporting Table S3: GC‐IMS analysis conditions.

Supporting Table S4: Gas chromatography conditions.

Supporting Table S5: GC‐IMS integration parameters of volatile compounds identified in N. tangutorum wine samples with different blending methods (corresponding to the serial number in Figure 4C.)

Supporting Table S6: Volatile compounds’ OAVs among *N. tangutorum* wine samples with different blending methods.

Supporting Table S7: Volatile compounds’ OAVs among N. tangutorum wine samples with different blending methods (OAV > 1).

## Supporting information


**Supporting Information** Additional supporting information can be found online in the Supporting Information section.

## Data Availability

The data supporting the findings of this study are available from the corresponding author upon reasonable request.
