# Supplementary material for: Optimizing Sensory Experience and Aroma Profile Through Novel Blending: A Dual GC‐MS/GC‐IMS Approach to Characterize Nitraria tangutorum Bobrov.–Blended Wine
Source: Int J Food Sci. 2026 Apr 29;2026:3378651. doi: 10.1155/ijfo/3378651 (PMC13128985; doi:10.1155/ijfo/3378651)
Supplement: Supplementary file 1 — Supporting Information Additional supporting information can be found online in the Supporting Information section. [file IJFO-2026-3378651-s001.docx]

**Supplementary Table S1**

Soluble solids and titratable acid contents in the first three raw materials prior to fermentation.

| Berries | *N. tangutorum* | Meili | ‘Shine Muscat’ |
| --- | --- | --- | --- |
| Total soluble solids/^○^Brix | 230.371 | 198 | 175.1 |
| Titratable acidity (g/L) | 3.496 | 5.81 | 4.11 |

**Supplementary Table S2**

Overall sensory evaluation criteria of *N. tangutorum* blended wine

| Project | Grade | Sensory scale |
| --- | --- | --- |
| Appearance | 0~4 | The color is light purple red or dark purple red, the wine is opaque, and the turbidity and foam are obvious. |
|  | 5~6 | The color is light purple red, the transparency of the wine is poor, and there is a slight turbidity and foam. |
|  | 7~ 10 | Purple red or ruby red, the wine body is more transparent, no obvious turbidity and foam. |
|  | 10~ 15 | Purple red or ruby red, shiny, transparent, clear, no turbidity and foam, eye-pleasing coordination |
| Aroma | 0~4 | The aroma is weak, and the odor is obvious. |
|  | 5~ 14 | Fruity and wine aroma is moderate, slightly peculiar smell. |
|  | 15~30 | The aroma is strong and pleasant and harmonious, mellow, rich and harmonious aroma, pure and elegant wine aroma, no peculiar smell. |
|  | 0~7 | Poor balance, taste too sweet or spicy too strong, fresh mellow thickness difference. |
| Taste | 8~ 12 | The balance is poor, the taste is sour or sweet and greasy, the spicy burning feeling is strong, and the mellow thickness is general. |
|  | 13~27 | The balance is better, the sweet and sour is general, the spicy burning sensation is moderate, the fresh mellow is thicker, and the palatability is softer. |
|  | 28~44 | Good balance, sweet and sour palatability, fresh and mellow, palatable and soft. |
| Style | 0~7 | Does not have the style of white thorn wine |
|  | 8~9 | The style is not prominent, the wine body is more coordinated. |
|  | 10~ 11 | *N. tangutorum* wine style is obvious, wine body coordination |

**Supplementary Table S3**

GC-IMS Analysis Conditions

| Gas chromatography-ion mobility spectrometry unit | |
| --- | --- |
| Analysis time | 30 min |
| Chromatographic column type | Producing area : RESTEK Company, USA ; chromatographic column polarity : WAX ; the column length was 30 m, the inner diameter was ID−0.53 mm, and the film thickness was FT − 1 μm. |
| Column temperature | 60 ℃ |
| Carrier gas/drift gas | N_2_ |
| IMS temperature | 45 ℃ |
| Automatic headspace sampling unit | |
| Sample volume | \ |
| Incubation period | 10 min |
| Brooding temperature | \ |
| Needle temperature | 85 ℃ |
| Incubation speed | 500 rpm |

**Supplementary Table S4**

Gas Chromatography Conditions

| **GC-IMS condition** | | | |
| --- | --- | --- | --- |
| Time | E1 | E2 | R |
| 00:00,000 | 150mL/min | 2 ml/min | rec |
| 02:00,000 | 150mL/min | 2 ml/min | - |
| 10:00,000 | 150mL/min | 10 ml/min | - |
| 20:00,000 | 150mL/min | 100 ml/min | - |
| 30:00,000 | 150mL/min | 100 ml/min | stop |

**Supplementary Table S5**

GC-IMS integration parameters of volatile compounds identified in *N. tangutorum* wine samples with different blending methods. (Corresponding to the serial number in Fig. 4C above.)

| Count | Compound | CAS# | Formula | MW | RI | Rt [sec] | Dt [RIPrel] |
| --- | --- | --- | --- | --- | --- | --- | --- |
| 1 | Pentan-2-one | C107879 | C5H10O | 86.1 | 1001.9 | 367.404 | 1.37323 |
| 2 | 2-Methyl-1-propanol | C78831 | C4H10O | 74.1 | 1103.9 | 486.223 | 1.36115 |
| 3 | 3-Methyl-1-butanol | C123513 | C5H12O | 88.1 | 1215.7 | 677.271 | 1.49004 |
| 4 | Acetic acid monomer | C64197 | C2H4O2 | 60.1 | 1498.4 | 1188.062 | 1.04814 |
| 5 | Acetic acid dimer | C64197 | C2H4O2 | 60.1 | 1497.1 | 1185.101 | 1.15159 |
| 6 | ethyl acetate | C141786 | C4H8O2 | 88.1 | 898.4 | 299.04 | 1.34332 |
| 7 | ethanol | C64175 | C2H6O | 46.1 | 945.7 | 328.055 | 1.12947 |
| 8 | acetone | C67641 | C3H6O | 58.1 | 840.9 | 267.124 | 1.11788 |
| 9 | 2-Methyl propanal monomer | C78842 | C4H8O | 72.1 | 836.1 | 264.637 | 1.0947 |
| 10 | 2-Methyl propanal dimer | C78842 | C4H8O | 72.1 | 833.7 | 263.394 | 1.28432 |
| 11 | dimethyl sulfide | C75183 | C2H6S | 62.1 | 801.8 | 247.406 | 0.95862 |
| 12 | methyl acetate | C79209 | C3H6O2 | 74.1 | 853.9 | 274.034 | 1.19743 |
| 13 | Propan-2-ol | C67630 | C3H8O | 60.1 | 926.4 | 315.877 | 1.2133 |
| 14 | Propanol dimer | C71238 | C3H8O | 60.1 | 1051.4 | 420.241 | 1.24733 |
| 15 | 1-Penten-3-one | C1629589 | C5H8O | 84.1 | 1026.3 | 392.521 | 1.07823 |
| 16 | Butanol | C71363 | C4H10O | 74.1 | 1154.7 | 572.802 | 1.3759 |
| 17 | 1-Penten-3-ol | C616251 | C5H10O | 86.1 | 1171.7 | 605.224 | 0.94324 |
| 18 | Ethyl 3-methylbutanoate | C108645 | C7H14O2 | 130.2 | 1083.3 | 458.157 | 1.66039 |
| 19 | Ethyl butanoate | C105544 | C6H12O2 | 116.2 | 1054.4 | 423.654 | 1.56574 |
| 20 | Isoamyl acetate | C123922 | C7H14O2 | 130.2 | 1138.8 | 544.109 | 1.75212 |
| 21 | Ethyl lactate | C97643 | C5H10O3 | 118.1 | 1355.3 | 892.95 | 1.54177 |
| 22 | acetaldehyde monomer | C75070 | C2H4O | 44.1 | 735.1 | 217.085 | 0.95701 |
| 23 | acetaldehyde dimer | C75070 | C2H4O | 44.1 | 736.1 | 217.478 | 1.01703 |
| 24 | ethyl propanoate | C105373 | C5H10O2 | 102.1 | 975.4 | 347.754 | 1.45758 |
| 25 | isobutyl acetate | C110190 | C6H12O2 | 116.2 | 1030.7 | 397.261 | 1.62058 |
| 26 | Octanoic acid ethyl ester | C106321 | C10H20O2 | 172.3 | 1456.4 | 1092.552 | 1.47579 |
| 27 | butyl acetate monomer | C123864 | C6H12O2 | 116.2 | 1066.7 | 438.02 | 1.23888 |
| 28 | butyl acetate dimer | C123864 | C6H12O2 | 116.2 | 1065.4 | 436.457 | 1.61568 |
| 29 | octanal | C124130 | C8H16O | 128.2 | 1293 | 788.643 | 1.40256 |

**Supplementary Table S6**

Volatile compounds’ OAVs among *N. tangutorum* wine samples with different blending methods.

| Compound | Threhold (mg/L) | CK | ML | QK-SM | NT-ML-2 | NT-QK-SM-2 |
| --- | --- | --- | --- | --- | --- | --- |
| β-Phenethyl acetate | 0.21 | 0.30 ± 0.00c | 0.76 ± 0.01a | 0.34 ± 0.03c | 0.60 ± 0.01b | 0.30 ± 0.00c |
| Hexyl acetate | 0.67 | 0.55 ± 0.03b | 0.34 ± 0.07d | 1.10 ± 0.17a | 0.45± 0.02c | 0.17 ± 0.00e |
| Ethyl succinate | 200 | 0.00 ± 0.00b | 0.00 ± 0.00b | 0.01 ± 0.00a | 0.00 ± 0.00b | 0.01 ± 0.00a |
| Ethyl 2-methylbutanoate | 0.018 | 0.37 ± 0.02b | 0.00 ± 0.00d | 0.33 ± 0.10c | 0.30 ± 0.02c | 0.65 ± 0.02a |
| Ethyl 2-hydroxybenzoate | 15.781 | 0.12 ± 0.00a | 0.11 ± 0.00a | 0.11 ± 0.00a | 0.12 ± 0.00a | 0.11 ± 0.00a |
| Ethyl 3-methylbutanoate | 0.003 | 11.44 ± 0.11a | 7.33 ± 1.15c | 11.56 ± 0.67a | 4.56 ± 0.11d | 10.56 ± 0.11b |
| Ethyl dodecanoate | 1.5 | 0.03 ± 0.00b | 0.04± 0.00a | 0.02 ± 0.00c | 0.03 ± 0.00b | 0.03 ± 0.00b |
| Ethyl acetate | 7.5 | 16.13 ± 0.00e | 125.31± 0.00a | 96.93 ± 0.00c | 102.45 ± 0.00b | 71.33 ± 0.00d |
| Ethyl nonanoater | 1.3 | 0.01 ± 0.00b | 0.02 ± 0.00a | 0.00 ± 0.00c | 0.01± 0.00b | 0.01 ± 0.00b |
| Ethyl caproate | 0.014 | 10.09 ± 0.02e | 57.50 ± 3.01c | 110.24 ± 8.31a | 43.95 ± 0.02d | 77.55 ± 0.02b |
| Isobutyl acetate | 1.6 | 0.07 ± 0.00c | 0.19 ± 0.01a | 0.00 ± 0.00e | 0.14 ± 0.00b | 0.03 ± 0.00d |
| Isopentyl hexanoate | 0.05 | 0.13 ± 0.01b | 0.18 ± 0.01a | 0.21 ± 0.02a | 0.13 ± 0.01b | 0.13 ± 0.01b |
| Methyl salicylate | 0.04 | 0.03 ± 0.00a | 0.00 ± 0.00b | 0.00 ± 0.00b | 0.03 ± 0.00a | 0.03 ± 0.00a |
| Methyl caprylate | 0.005 | 1.73 ± 0.07c | 2.40 ± 0.12a | 1.40 ± 0.12d | 2.07 ± 0.07b | 1.67 ± 0.07cd |
| Ethyl lactate | 157.81 | 0.18 ± 0.00c | 0.07 ± 0.00d | 0.73 ± 0.05a | 0.09 ± 0.00d | 0.54 ± 0.00b |
| Ethyl butyrate | 0.035 | 0.70 ± 0.01d | 10.18 ± 0.53c | 51.39 ± 3.87a | 5.92 ± 0.01cd | 39.52 ± 0.01b |
| Isoamyl acetate | 0.03 | 15.62 ± 0.01c | 206.58 ± 10.85a | 5.13 ± 0.38c | 138.81 ± 5.01b | 8.94 ± 0.01c |
| 1-Butanol | 150 | 0.01 ± 0.00d | 0.03 ± 0.00b | 0.06 ± 0.01a | 0.02 ± 0.00c | 0.03 ± 0.00b |
| 3-Methyl-1-butanol | 30 | 1.75 ± 0.00d | 9.33 ± 0.49a | 3.01 ± 0.23c | 6.99 ± 0.00b | 2.28 ± 0.00cd |
| 4-Methyl-1-pentanol | 50 | 0.00 ± 0.00 | 0.00 ± 0.00 | 0.00 ± 0.00 | 0.00 ± 0.00 | 0.00 ± 0.00 |
| 1-Decanol | 0.4 | 0.36 ± 0.00a | 0.39 ± 0.02a | 0.42 ± 0.03a | 0.37 ± 0.00a | 0.36 ± 0.00a |
| 1-Heptanol | 2.5 | 0.01 ± 0.00e | 0.96 ± 0.05a | 0.43 ± 0.03c | 0.69 ± 0.00b | 0.19 ± 0.00d |
| 1-Hexanol | 8 | 0.03 ± 0.00c | 0.04 ± 0.00b | 0.06 ± 0.01a | 0.03 ± 0.00c | 0.05 ± 0.00b |
| 2-Ethyl-1-hexanol | 8 | 0.00 ± 0.00 | 0.00 ± 0.00 | 0.00 ± 0.00 | 0.00 ± 0.00 | 0.00 ± 0.00 |
| 1-Octanol | 0.04 | 0.54 ± 0.01c | 1.49 ± 0.08a | 0.84 ± 0.06b | 0.89 ± 0.01b | 0.64 ± 0.01c |
| 1-Octen-3-ol | 0.001 | 14.67 ± 0.33d | 82.67 ± 4.33a | 50.67 ± 3.76b | 53.33 ± 0.33b | 25.33 ± 0.33c |
| 1-Pentanol | 1 | 0.00 ± 0.00d | 11.48 ± 0.60b | 21.35 ± 1.61a | 5.70 ± 0.00c | 11.06 ± 0.00b |
| 1-Propanol | 306 | 0.17 ± 0.00d | 0.22 ± 0.01bc | 0.30 ± 0.02a | 0.20 ± 0.00cd | 0.24 ± 0.00b |
| 2-Methyl-1-propanol | 40 | 0.68 ± 0.00d | 4.47 ± 0.24a | 1.58 ± 0.12c | 2.54 ± 0.00b | 0.85 ± 0.00d |
| 3-methylthiopropanol | 0.5 | 0.71 ± 0.00c | 14.86 ± 0.78a | 1.26 ± 0.10c | 7.00 ± 0.00b | 0.73 ± 0.00c |
| (E)-2-Hexen-1-ol | 0.4 | 0.11 ± 0.00a | 0.02 ± 0.00d | 0.03 ± 0.00c | 0.03 ± 0.00c | 0.05 ± 0.00b |
| (Z)-2-Hexen-1-ol | 0.4 | 1.61 ± 0.00e | 86.80 ± 4.56a | 26.92 ± 2.03c | 50.40 ± 0.00b | 14.32 ± 0.00d |
| (E) -3-Hexen-1-ol | 0.4 | 0.05 ± 0.00c | 0.28 ± 0.01c | 4.21 ± 0.32a | 0.18 ± 0.00c | 1.53 ± 0.00b |
| (Z) -3-Hexen-1-ol | 0.4 | 0.57 ± 0.00a | 0.00 ± 0.00d | 0.00 ± 0.00d | 0.08 ± 0.00c | 0.15 ± 0.00b |
| Benzyl alcohol | 200 | 0.01 ± 0.00a | 0.00 ± 0.00b | 0.00 ± 0.00b | 0.00 ± 0.00b | 0.00 ± 0.00b |
| Phenylethyl Alcohol | 10 | 1.28 ± 0.01c | 2.77 ± 0.14a | 1.26 ± 0.10c | 2.52 ± 0.01b | 1.09 ± 0.01c |
| Isopentanoic acid | 0.033 | 13.69 ± 0.01a | 0.00 ± 0.00d | 3.73 ± 0.28c | 7.62 ± 0.01b | 7.22 ± 0.01b |
| Hexanoic acid | 3 | 1.04 ± 0.00a | 0.36 ± 0.02c | 0.39 ± 0.02bc | 0.43± 0.00b | 0.39 ± 0.00bc |
| n-Decanoic acid | 1 | 0.13 ± 0.00c | 0.20 ± 0.01a | 0.13 ± 0.01c | 0.16 ± 0.00b | 0.13 ± 0.00c |
| Isobutyric acid | 2.3 | 0.12 ± 0.00b | 0.13 ± 0.01ab | 0.14 ± 0.01a | 0.12 ± 0.00b | 0.12 ± 0.00b |
| Octanoic acid | 0.5 | 0.89 ± 0.00b | 1.97 ± 0.10a | 1.00 ± 0.08b | 1.79 ± 0.00a | 0.87 ± 0.00b |
| Decanal | 0.001 | 1.00 ± 0.00b | 0.00 ± 0.00c | 0.00 ± 0.00c | 1.00 ± 0.00b | 1.33 ± 0.33a |
| Furfural | 14.1 | 0.01 ± 0.00c | 0.01 ± 0.00c | 0.05 ± 0.00b | 0.01 ± 0.00c | 1.09 ± 0.00a |
| Nonanal | 0.001 | 13.33 ± 0.33b | 13.00 ± 0.58b | 17.33 ± 1.45a | 13.67 ± 0.33b | 14.33 ± 0.33b |
| Farnesol | 0.02 | 2.32 ± 0.02a | 2.37 ± 0.13a | 2.53 ± 0.19a | 2.37 ± 0.02a | 2.38 ± 0.02a |
| α-Terpineol | 0.033 | 0.77 ± 0.01a | 0.33 ± 0.02c | 0.45 ± 0.04b | 0.38 ± 0.01c | 0.23 ± 0.01d |
| Citronellol | 0.01 | 1.17 ± 0.03ab | 1.10 ± 0.06ab | 1.03 ± 0.09b | 1.23 ± 0.03a | 1.17 ± 0.03ab |
| Geraniol | 0.03 | 0.89 ± 0.01a | 0.59 ± 0.03b | 0.77 ± 0.06c | 0.54 ± 0.01b | 0.52 ± 0.01b |
| Linalool | 0.025 | 0.75 ± 0.01b | 0.61 ± 0.04b | 1.05 ± 0.08a | 0.71 ± 0.01b | 0.67 ± 0.01b |
| 4-Ethylphenol | 0.44 | 0.15 ± 0.00a | 0.03 ± 0.00c | 0.03 ± 0.00c | 0.06 ± 0.00b | 0.05 ± 0.00b |
| β-Ionone | 0.1 | 0.05 ± 0.00a | 0.06 ± 0.00a | 0.00 ± 0.00b | 0.05 ± 0.00a | 0.00 ± 0.00b |

Note: Different letters indicate significant differences between different grapes (Tukey's test, P < 0.05).

**Supplementary Table S7**

Volatile compounds’ OAVs among *N. tangutorum* wine samples with different blending methods. (OAV>1).

| Compound | Threhold (mg/L) | CK | ML | QK-SM | NT-ML-2 | NT-QK-SM-2 |
| --- | --- | --- | --- | --- | --- | --- |
| Ethyl 3-methylbutanoate | 0.003 | 11.44 ± 0.11a | 7.33 ± 1.15c | 11.56 ± 0.67a | 4.56 ± 0.11d | 10.56 ± 0.11b |
| Ethyl acetate | 7.5 | 16.13 ± 0.00e | 125.31± 0.00a | 96.93 ± 0.00c | 102.45 ± 0.00b | 71.33 ± 0.00d |
| Ethyl caproate | 0.014 | 10.09 ± 0.02e | 57.50 ± 3.01c | 110.24 ± 8.31a | 43.95 ± 0.02d | 77.55 ± 0.02b |
| Methyl caprylate | 0.005 | 1.73 ± 0.07c | 2.40 ± 0.12a | 1.40 ± 0.12d | 2.07 ± 0.07b | 1.67 ± 0.07cd |
| Ethyl butyrate | 0.035 | 0.70 ± 0.01d | 10.18 ± 0.53c | 51.39 ± 3.87a | 5.92 ± 0.01cd | 39.52 ± 0.01b |
| Isoamyl acetate | 0.03 | 15.62 ± 0.01c | 206.58 ± 10.85a | 5.13 ± 0.38c | 138.81 ± 5.01b | 8.94 ± 0.01c |
| 3-Methyl-1-butanol | 30 | 1.75 ± 0.00d | 9.33 ± 0.49a | 3.01 ± 0.23c | 6.99 ± 0.00b | 2.28 ± 0.00cd |
| 1-Octen-3-ol | 0.001 | 14.67 ± 0.33d | 82.67 ± 4.33a | 50.67 ± 3.76b | 53.33 ± 0.33b | 25.33 ± 0.33c |
| 1-Pentanol | 1 | 0.00 ± 0.00d | 11.48 ± 0.60b | 21.35 ± 1.61a | 5.70 ± 0.00c | 11.06 ± 0.00b |
| 2-Methyl-1-propanol | 40 | 0.68 ± 0.00d | 4.47 ± 0.24a | 1.58 ± 0.12c | 2.54 ± 0.00b | 0.85 ± 0.00d |
| 3-methylthiopropanol | 0.5 | 0.71 ± 0.00c | 14.86 ± 0.78a | 1.26 ± 0.10c | 7.00 ± 0.00b | 0.73 ± 0.00c |
| (Z)-2-Hexen-1-ol | 0.4 | 1.61 ± 0.00e | 86.80 ± 4.56a | 26.92 ± 2.03c | 50.40 ± 0.00b | 14.32 ± 0.00d |
| (E) -3-Hexen-1-ol | 0.4 | 0.05 ± 0.00c | 0.28 ± 0.01c | 4.21 ± 0.32a | 0.18 ± 0.00c | 1.53 ± 0.00b |
| Phenylethyl Alcohol | 10 | 1.28 ± 0.01c | 2.77 ± 0.14a | 1.26 ± 0.10c | 2.52 ± 0.01b | 1.09 ± 0.01c |
| Isopentanoic acid | 0.033 | 13.69 ± 0.01a | 0.00 ± 0.00d | 3.73 ± 0.28c | 7.62 ± 0.01b | 7.22 ± 0.01b |
| Octanoic acid | 0.5 | 0.89 ± 0.00b | 1.97 ± 0.10a | 1.00 ± 0.08b | 1.79 ± 0.00a | 0.87 ± 0.00b |
| Decanal | 0.001 | 1.00 ± 0.00b | 0.00 ± 0.00c | 0.00 ± 0.00c | 1.00 ± 0.00b | 1.33 ± 0.33a |
| Furfural | 14.1 | 0.01 ± 0.00c | 0.01 ± 0.00c | 0.05 ± 0.00b | 0.01 ± 0.00c | 1.09 ± 0.00a |
| Nonanal | 0.001 | 13.33 ± 0.33b | 13.00 ± 0.58b | 17.33 ± 1.45a | 13.67 ± 0.33b | 14.33 ± 0.33b |
| Farnesol | 0.02 | 2.32 ± 0.02a | 2.37 ± 0.13a | 2.53 ± 0.19a | 2.37 ± 0.02a | 2.38 ± 0.02a |
| Citronellol | 0.01 | 1.17 ± 0.03ab | 1.10 ± 0.06ab | 1.03 ± 0.09b | 1.23 ± 0.03a | 1.17 ± 0.03ab |

Note: Different letters indicate significant differences between different grapes (Tukey's test, P < 0.05).
